# Patientenorientierte Lehrformate in der Klinischen Pharmazie – zum Status quo in der deutschen universitären Apothekerausbildung

**DOI:** 10.1007/s00103-025-04036-2

**Published:** 2025-03-20

**Authors:** Frank Dörje, Mirjam Gnadt, Jacqueline Bauer, Monika Dircks

**Affiliations:** 1 Apotheke des Universitätsklinikums Erlangen, Palmsanlage 3, 91054 Erlangen, Deutschland; 2 Department Chemie und Pharmazie, Friedrich-Alexander-Universität Erlangen-Nürnberg (FAU), Erlangen, Deutschland

**Keywords:** Klinische Pharmazie, Patientenorientierte Lehrformate, Fallbasiertes Lernen, Interprofessionelles Lernen, Apothekerausbildung, Clinical pharmacy, Patient-oriented teaching formats, Case-based learning, Interprofessional learning, Pharmacist education

## Abstract

Durch den Wandel des Berufsbildes von Apothekern in der Krankenhaus- und öffentlichen Apotheke in Deutschland stehen der Patient und seine individuelle Pharmakotherapie mehr denn je im Mittelpunkt der Pharmazie. Die Lehre im Fach Klinische Pharmazie soll angehende Apotheker dazu befähigen, die Arzneimitteltherapie des Patienten zu optimieren. Dazu sollen u. a. patientenorientierte Lehrformate eingesetzt werden. Mithilfe einer strukturierten Literaturrecherche und einer Ad-hoc-Umfrage an allen 22 deutschen Universitätsstandorten (Rücklaufquote 86 %, 19/22) wurde das Angebot an patientenorientierten Lehrformaten im Fach Klinische Pharmazie erhoben.

Es zeigt sich, dass zumindest das geforderte fallbasierte, theoretische patientenorientierte Lehren und Lernen gemäß Ausbildungsstandard 6 der Fachgruppe Klinische Pharmazie der Deutschen Pharmazeutischen Gesellschaft e. V. (DPhG) nahezu flächendeckend an den Universitätsstandorten (18/19) umgesetzt wird. 6 von 19 Ausbildungsstandorten bieten 4 oder mehr patientenorientierte Lehrformate an. Daneben werden die Formate „Bedside Teaching“ (12/19) und interprofessionelle Lehre (10/19) häufig praktiziert (mehrheitlich optional). Das „Bedside Teaching“ wird vielfach ehrenamtlich oder durch Lehraufträge von Krankenhausapothekern geleistet. Der zeitliche Umfang ist aufgrund stark begrenzter personeller Ressourcen deutlich limitiert. Das Gesamtbild zeigt eine sehr heterogen praktizierte patientenorientierte Lehre im Fach Klinische Pharmazie. Dies betrifft die Art des Lehrangebotes, den zeitlichen Umfang sowie den Anteil an Pflichtveranstaltungen. Die Novellierung der universitären Apothekerausbildung sollte zeitlich deutlich umfassendere und verpflichtende patientenorientierte Lehrformate im Fach Klinische Pharmazie vorsehen. Hierzu ist auch die adäquate Ausstattung mit klinisch kompetentem Lehrpersonal notwendig.

**Zusatzmaterial online:**

Zusätzliche Informationen sind in der Online-Version dieses Artikels (10.1007/s00103-025-04036-2) enthalten.

## Abstract (en)

Due to the change in the professional profile of pharmacists in hospital and public pharmacies in Germany, patients and their individual pharmacotherapy are more than ever within the focus of pharmacy. Teaching in clinical pharmacy should enable future pharmacists to optimize drug therapy for patients. For this purpose, inter alia, patient-oriented teaching formats should be employed. Using a structured literature search and an ad hoc survey among all 22 German pharmacy universities (response rate 86%, 19/22), the range of patient-oriented teaching formats in clinical pharmacy was assessed.

At a minimum, the required case-based, theoretical patient-oriented teaching and learning in accordance with the education standard 6 of the Clinical Pharmacy Section of the German Pharmaceutical Society (DPhG) is implemented among almost all sites (18/19). Six out of 19 educational sites offer four or more patient-oriented teaching formats. Furthermore, the formats bedside teaching (12/19) and interprofessional teaching (10/19) are frequently practiced (mostly optional). Bedside teaching is often provided on a voluntary basis or through teaching assignments by clinical pharmacists. The time available for patient-oriented teaching is clearly limited due to the very restricted personnel resources. The overall view shows that patient-oriented teaching in clinical pharmacy is practiced very heterogeneously. This applies to the type of courses offered, the temporal scope, and the proportion of compulsory courses. The amendment of university pharmacist education should provide significantly more comprehensive and mandatory patient-oriented teaching formats in the subject of clinical pharmacy. This also requires an adequate supply of clinically competent teaching staff.

## Hintergrund

Die erste Definition der „Klinischen Pharmazie“ des „Committee of Curriculum“ der „American Association of Colleges of Pharmacy“ (AACP) von 1968 lautete: „Clinical Pharmacy is that area within the pharmacy curriculum which deals with patient care with emphasis on drug therapy. Clinical pharmacy seeks to develop a patient-oriented attitude. Acquisition of new knowledge is secondary to attainment of skills in interprofessional and patient communication“ [1]. Ziel der Klinischen Pharmazie ist es, patientenorientierte pharmazeutische Dienstleistungen zu erbringen, die für eine sichere und wirksame Therapie mit Arzneimitteln notwendig sind [1].

Mit der Novellierung der Approbationsordnung für Apotheker im Oktober 2001 wurde der Bedeutung des Faches Klinische Pharmazie als damals neues und 5. Lehr- und Prüfungsfach in der universitären Apothekerausbildung auch in Deutschland Rechnung getragen [2].

Die Fachgruppe Klinische Pharmazie der Deutschen Pharmazeutischen Gesellschaft e. V. (DPhG) formulierte 2004 10 Standards zur Gestaltung der Pflichtveranstaltungen und Prüfungen im Fach Klinische Pharmazie. Als Oberziel der universitären Ausbildung im Fach Klinische Pharmazie wurde formuliert: „Die Studierenden sollen befähigt werden, die Gesamtsituation des Patienten hinsichtlich seiner Erkrankung und Arzneimitteltherapie zu verstehen und diese Kenntnisse einzusetzen, um Patienten und Ärzte sowie Angehörige anderer Gesundheitsberufe in der optimalen Arzneimittelanwendung evidenzbasiert und verantwortlich zu unterstützen“ [3].

Das Berufsbild des Apothekers in der Krankenhaus- und öffentlichen Apotheke hat sich in Deutschland in den letzten 2 Jahrzehnten stark gewandelt. Mehr denn je stehen der Patient und seine individuelle Pharmakotherapie im Mittelpunkt der Pharmazie. Mit der Änderung der Apothekenbetriebsordnung im Juni 2012 wurde das Medikationsmanagement in den Katalog der pharmazeutischen Tätigkeiten aufgenommen [4]. Im Perspektivpapier „Apotheke 2030“ der ABDA – Bundesvereinigung Deutscher Apothekerverbände e. V. von 2014 sind patientenorientierte Dienstleistungen (z. B. Durchführung von Medikationsanalysen, systematisches Medikationsmanagement) als zentrales Element zur Schärfung des heilberuflichen Profils genannt. Dadurch können Apotheker eine zentrale Rolle zur Verbesserung der Arzneimitteltherapiesicherheit (AMTS) einnehmen und gleichzeitig die Netzwerkzusammenarbeit mit der Ärzteschaft und anderen Gesundheitsberufen stärken [5]. Im Grundsatzpapier der ABDA von 2014 zur Medikationsanalyse und zum Medikationsmanagement wurden grundlegende Definitionen und Festlegungen zur Umsetzung formuliert [6]. Inzwischen wurden in Deutschland honorierte, patientenorientierte pharmazeutische Dienstleistungen (z. B. erweiterte Medikationsberatung bei Polymedikation) im Vor-Ort-Apothekenstärkungsgesetz (VOASG) für öffentliche Apotheken ermöglicht und 2022 de facto eingeführt [7].

Im stationären Versorgungsbereich nimmt der klinisch-pharmazeutische Einsatz von Stationsapothekern in Deutschland im letzten Jahrzehnt stetig an Bedeutung und Umfang zu [8–10]. Zudem führen immer mehr Krankenhäuser das auch vom Bundesverband Deutscher Krankenhausapotheker e. V. (ADKA) geforderte systematische „Closed Loop Medication Management“ ein, das ein Medikationsmanagement durch klinische Apotheker obligat vorsieht [11, 12].

Die Fachgruppe Klinische Pharmazie der DPhG schreibt in der Präambel zu den fortgeschriebenen Standards zur universitären Ausbildung im Fach Klinische Pharmazie 2015: „Die für diese Neuausrichtung der Apothekeraufgaben und -verantwortlichkeiten erforderlichen Kenntnisse und Fähigkeiten werden im Pharmaziestudium vor allem im Fach Klinische Pharmazie vermittelt, das jedoch an den einzelnen Hochschulstandorten in unterschiedlichem Umfang und mit divergierenden Inhalten gelehrt wird“ [3, 13]. Dabei wird betont, dass für die Übernahme von patientenorientierten, pharmazeutischen Dienstleistungen flächendeckende, hochqualitative Lehrveranstaltungen notwendig sind. Dazu werden in den Standards u. a. das fallbezogene Lernen sowie das Lernen im direkten Patientenkontakt gefordert (Tab. 1). Pudritz und Wahl-Schott konstatierten 2019 in ihrem Grundlagenbeitrag, dass die Universitätsstandorte bundesweit in der Pharmazie sehr unterschiedliche Pflicht- und optionale Lehrveranstaltungen mit sehr heterogenen Möglichkeiten zur klinischen Praxiserfahrung mit Patientenbeteiligung anbieten [14].

**Tab. 1:** Ausgewählte Standards mit Bezug zu patientenorientierten Lehrformaten in der universitären Ausbildung im Fach Klinische Pharmazie, entwickelt von der Fachgruppe Klinische Pharmazie der Deutschen Pharmazeutischen Gesellschaft e. V. (DPhG; [13])

Standard-Nummer	Beschreibung
5	Das Seminar „Klinische Pharmazie“ wird zum überwiegenden Teil in kleineren Gruppen durchgeführt, um interaktives Lernen zu ermöglichen.
6	Bei den im Rahmen des Seminars „Klinische Pharmazie“ zu bearbeitenden Beispielen spielen fallbasierte Übungen eine zentrale Rolle.
7	Während der Lehrveranstaltungen der Klinischen Pharmazie besteht ein direkter Kontakt mit Patienten.
8	In die Ausbildung im Fach Klinische Pharmazie sind sogenannte Teacher/Practitioner fest eingebunden.
10	Im Rahmen der Lehrveranstaltungen zur Klinischen Pharmazie werden schriftliche Ausarbeitungen angefertigt.
11	Im Rahmen der Lehrveranstaltungen zur Klinischen Pharmazie werden auch Grundlagen der Kommunikation mit Patienten und anderen Berufsgruppen im Gesundheitswesen thematisiert.

Die skizzierten beruflich stark veränderten Anforderungen an den Apothekerberuf in der Offizin- und Krankenhausapotheke führten dazu, dass die Bundesapothekerkammer gemeinsam mit einer Vielzahl von Verbänden und Vereinigungen in einem „Runden-Tisch“-Verfahren ein Positionspapier zur Novellierung der Approbationsordnung für Apotheker erarbeitete und im Jahr 2022 veröffentlichte ([15, 16]; siehe auch Beitrag von Winter in diesem Themenheft). Zu den erarbeiteten Positionen zählt die Erkenntnis, dass die Fächer Klinische Pharmazie und Pharmakologie zeitlich und inhaltlich im Studium erweitert werden sollen, um die benötigten, vertieften Kenntnisse und Kompetenzen zur Ausübung einer patientenorientierten Klinischen Pharmazie zu erlangen. Dabei soll stärker als bisher fallbezogen gelehrt werden und eine verstärkte interprofessionelle Lehre insbesondere zwischen der Pharmazie und der Humanmedizin angestrebt werden [15].

Im Kontext dieses aktuellen Entwicklungsstandes und vor dem Hintergrund der Diskussion zur notwendigen Novellierung der Approbationsordnung für Apotheker verfolgt die vorliegende Übersichtsarbeit das Ziel, die Art und den Umfang von patientenorientierten Lehrformaten zu beschreiben, die im Fach Klinische Pharmazie in der universitären Ausbildung für Pharmaziestudierende in Deutschland angeboten werden.

## Methodisches Vorgehen

Um die verschiedenen patientenorientierten Lehrformate, die in Deutschland angeboten werden, zu identifizieren, wurde eine strukturierte Literaturrecherche durchgeführt. Dabei wurden veröffentlichte Arbeiten aus Deutschland berücksichtigt und die im Onlinematerial 1 genannten Suchbegriffe und Quellen genutzt.

Um den derzeitigen Einsatz patientenorientierter Lehrformate im Fach Klinische Pharmazie zu evaluieren, wurde vom 27.10. bis zum 15.11.2024 eine Ad-hoc-Befragung unter den Lehrverantwortlichen für das Fach Klinische Pharmazie an allen pharmazeutischen Universitätsstandorten in Deutschland durchgeführt. Im Fragebogen (siehe Onlinematerial 2) wurden die mittels strukturierter Literaturrecherche identifizierten Lehrformate abgefragt. Die Umfrage umfasste u. a. den zeitlichen Umfang des angebotenen Lehrformates, die eingesetzten Lehrpersonen für die Betreuung der Studierenden und die Fragestellung, ob es sich um eine Pflicht- oder optionale Zusatzveranstaltung handelt.

Im Folgenden sollen zunächst die Ergebnisse der Ad-hoc-Umfrage beleuchtet werden und anschließend die einzelnen Lehrformate inhaltlich beschrieben werden sowie pointiert Bezug auf Umfrageergebnisse genommen werden.

## Ad-hoc-Umfrage unter allen deutschen Universitätsstandorten zum Status quo des Einsatzes von patientenorientierten Lehrformaten im Fach Klinische Pharmazie

**Abb. 1:**
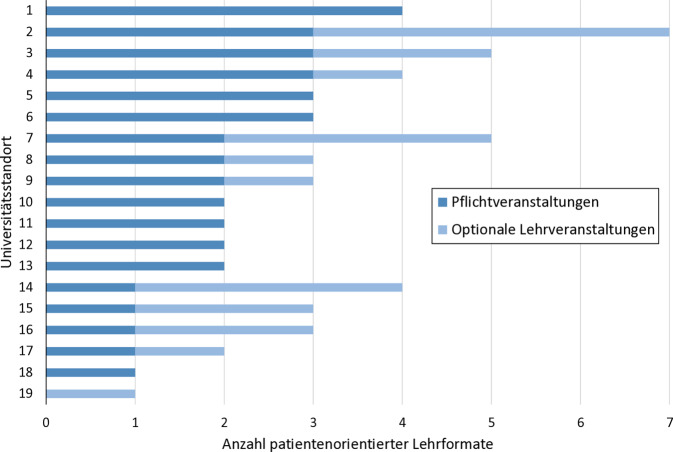
Anzahl der patientenorientierten Lehrformate an den einzelnen Universitätsstandorten gegliedert in Pflichtveranstaltungen und optionale Lehrveranstaltungen. (Quelle: eigene Abbildung)

86 % (19/22) der angeschriebenen deutschen Universitätsstandorte sendeten den ausgefüllten Fragebogen fristgemäß zurück. Auffällig ist die sehr heterogene Verteilung der Anzahl der angebotenen patientenorientierten Lehrveranstaltungen an den Universitätsstandorten. Während ein Standort 4 patientenorientierte Pflichtveranstaltungen durchführt, bietet ein Standort zurzeit keine einzige Pflichtveranstaltung an (Abb. 1). Unter Berücksichtigung der optionalen Lehrformate erhöht sich die Bandbreite von einer auf bis zu 7 verschiedene patientenorientierte Veranstaltungen pro Standort.

**Abb. 2:**
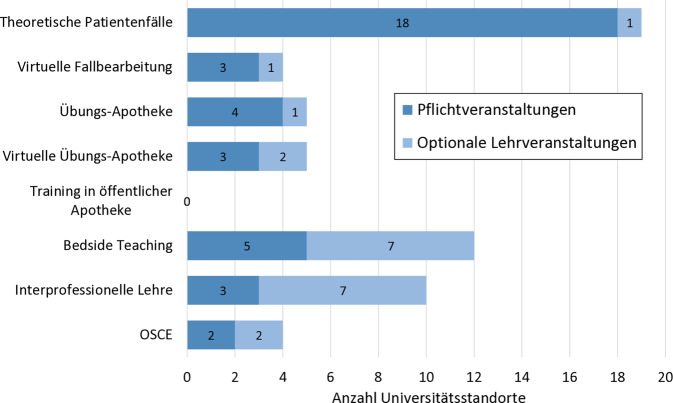
Patientenorientierte Lehrformate an den 19 Universitätsstandorten gegliedert in Pflichtveranstaltungen und optionale Lehrveranstaltungen; Abkürzung: *OSCE* „objective structured clinical examination“. (Quelle: eigene Abbildung)

An nahezu allen Universitäten (18 von 19) werden verpflichtend Fallbearbeitungen mit theoretischen Patientenfällen durchgeführt (Abb. 2). Der zeitliche Umfang dieser Pflichtveranstaltungen beträgt im Median 1,43 Semesterwochenstunden (SWS; min. 0,7, max. 8,0).

Die Mehrheit der antwortenden Standorte (12 von 19) bietet ein „Bedside-Teaching“-Praktikum trotz hohem organisatorischem und personellem Aufwand an (Abb. 2). Der Grund dürfte in der historisch engen Verknüpfung zwischen der Krankenhauspharmazie und dem universitären Fach Klinische Pharmazie liegen. Krankenhausapotheker engagieren sich seit der Etablierung des Faches in das Pharmaziestudium im Jahr 2001 und leisten vielerorts einen wertvollen Beitrag zur Ausbildung. Die Betreuung der „Bedside-Teaching“-Praktika wird gemäß Umfrageergebnis überwiegend von klinisch tätigen Apothekern übernommen. Allerdings handelt es sich bei den Pflicht- und optionalen Lehrveranstaltungen meist um einmalige Lehreinheiten, deren zeitlicher Umfang von 4 h bis zu 2 Nachmittagen reicht.

Die enge Verbindung zwischen Klinik und Universität erklärt das ebenfalls relativ häufig genannte Angebot der interprofessionellen Lehre an 10 von 19 Standorten (Abb. 2), da dieses Format meist im Krankenhaus unter Einbindung von Klinikärzten durchgeführt wird.

## Übersicht über patientenorientierte Lehrformate in der Klinischen Pharmazie

Im Mittelpunkt der Klinischen Pharmazie steht also nicht, wie in den klassischen Fächern des Pharmaziestudiums, das singuläre Arzneimittel, sondern der individuelle Patient und die Arzneimittelanwendung am Patienten und durch den Patienten, die angewandte Arzneimitteltherapie im klinischen Kontext und in ihrer Gesamtheit. Neben dem notwendigen Aufbau eines soliden klinisch-pharmazeutischen Fachwissens ist die Erlangung von patientenorientierten Kompetenzen und Fähigkeiten (z. B. Kommunikation) von entscheidender Bedeutung. Geeignet sind dazu verschiedene Patientenfall-orientierte Lehrformate mit und ohne direkten Patientenkontakt. Wie die Ergebnisse der Ad-hoc-Befragung und der strukturierten Literaturrecherche zeigen, werden an deutschen Universitätsstandorten vielseitige patientenorientierte Lehrformate eingesetzt. Diese werden im Folgenden synoptisch vorgestellt.

### Patientenorientierte Lehr- und Lernformate im universitären Setting ohne realen Patientenkontakt

#### „Analoge“ Patientenfallbearbeitung („Paper Case“).

Ein Beispiel für problemorientiertes Lernen im Rahmen des fallbezogenen Lernens ist das im Fach Klinische Pharmazie an der Friedrich-Alexander-Universität Erlangen-Nürnberg (FAU) durchgeführte Pflicht-Pharmakotherapieseminar. Dort bearbeiten die Pharmaziestudierenden in Kleingruppen über 2 Wochen 18 theoretische Patientenfälle aus dem ambulanten oder stationären Setting. Dazu lernen die Studierenden zunächst methodische Vorgehensweisen zur Durchführung einer Medikationsanalyse. Die Studierenden lernen selbstständig subjektive und objektive Patientendaten zu identifizieren, arzneimittelbezogene Probleme zu erkennen und Lösungsvorschläge zu entwickeln. Bei der Beurteilung der Medikation müssen die Studierenden eigenständig geeignete Quellen (z. B. Leitlinien) identifizieren und Arzneimittelinteraktionsprogramme anwenden. Mithilfe einer Musterpräsentation werden die Ergebnisse am folgenden Tag von einer Studierendengruppe im gesamten Semester vorgestellt und alternative Vorgehensweisen und Lösungsvorschläge kritisch diskutiert. Mithilfe dieses einfach zu etablierenden Patientenfall-orientierten Lehrformates wird also nicht nur gelerntes klinisch-pharmazeutisches Fachwissen durch Anwendung gefestigt, vielmehr müssen die Studierenden arzneimittelbezogene Probleme nach klinischer Relevanz priorisieren, sind aufgefordert, ihr Wissen zu reflektieren und Wissenslücken durch eigenständige Recherche zu schließen. Unsere Ad-hoc-Umfrage zeigt, dass der überwiegende Teil der Universitätsstandorte dieses Lehrformat als Pflichtveranstaltung in ihr Curriculum integriert haben, lediglich ein Standort bietet dieses Lehrformat nur optional als 3‑wöchiges Wahlpflichtfachpraktikum an (Abb. 2).

#### Virtuelle Patientenfallbearbeitung.

Die Möglichkeiten der virtuellen Darstellung und Bearbeitung eines Patientenfalls reichen von einfachen Patientenbeschreibungen mit anschließender Aufgabenstellung bis zur hochkomplexen Nachstellung der Realität, auch „virtual patient“ und „serious gaming“ genannt [17, 18]. An der Ludwigs-Maximilians-Universität (LMU) München werden im Fach Klinische Pharmazie optional vorlesungsbegleitende Fallbearbeitungen mit dem Programm Casus® angeboten. Dabei erhalten die Studierenden zunächst wie bei einem „Paper Case“ eine Patienten- und Situationsbeschreibung und werden dann schrittweise durch den Fall geführt. Bei jedem Schritt erhält der Studierende eine „Karte“ mit neuen Informationen und zu bearbeitende Fragen bzw. Aufgaben. Der vorbereitende Tutor kann in der Erstellung der Fälle aus unterschiedlichen Frage- und Antwortmöglichkeiten (z. B. Multiple-Choice‑, Zuordnungs- oder offene Fragen) wählen. Das digitale Format erlaubt direktes Feedback nach jeder Frage. Ein Vorteil dieses Formates ist, dass der Arbeitsaufwand (z. B. personelle Ressourcen) unabhängig von der Anzahl der Studierenden konstant bleibt. Ein Nachteil im Vergleich zum „Paper Case“ ist die geführte, nicht selbstständige Entscheidung über das Vorgehen bei der Bearbeitung des Falls [14]. 3 weitere Standorte nutzen aktuell verpflichtend eine virtuelle Patientenfallbearbeitung in der universitären Apothekerausbildung (Abb. 2). Bei einem Standort ist es in der konkreten Planung.

#### Übungsapotheke mithilfe von (Laien‑)Schauspielern.

Übungsapotheken sind an 4 Universitätsstandorten ein fester, verpflichtender und an einem ein optionaler Bestandteil des Lehrangebots im Fach Klinische Pharmazie (Abb. 2). Übungsapotheken, oder auch Trainingsapotheken, sind möglichst real nachgestellte Apotheken und dienen der Simulation von typischen Beratungssituationen. Die Studierenden sind aufgefordert, nach intensiver Auseinandersetzung mit einem Krankheitsbild einen „Patienten“, meist gespielt durch einen Kommilitonen, zu diesem Thema zu beraten [19, 20]. Im Medikations-Management-Center des Instituts für Pharmazie der Freien Universität Berlin steht darüber hinaus ein Raum für das „Brown-Bag-Verfahren“ zur Verfügung [21]. Hier bringt ein „Patient“ seine gesamten Medikamente zur Arzneimittelberatung in die Übungsapotheke mit.

Neben der Anwendung des Fachwissens steht bei der Simulation von Beratungsgesprächen vor allem das Erwerben von Kommunikationsfähigkeiten zum sicheren Führen von Patientengesprächen im Vordergrund. Durch das Üben in kleineren Gruppen können die Studierenden nicht nur durch Feedback vom Tutor, sondern auch durch gegenseitiges Beobachten und Bewerten voneinander lernen. Die Aufzeichnung mit einer Kamera fördert darüber hinaus die Auseinandersetzung mit den eigenen nonverbalen Kommunikationsfähigkeiten und das Erlernen einer guten Patientengesprächsführung.

#### Virtuelle Übungs-Offizin-Apotheke.

Um die Vorteile des digitalen Lernens zu nutzen, wird aktuell an 5 Universitätsstandorten das Programm MyDispense® genutzt (Abb. 2). Dargestellt wird eine virtuelle Apotheke mit üblicher Einrichtung (z. B. Beratungstresen, Computerprogramm, Kassensystem, Arzneimittelvorräte; [22]). Analog zur Realität kommt ein Patient mit einem bestimmten Anliegen in die virtuelle Apotheke, in der der Studierende virtuell die Rolle des Apothekers einnimmt und aus verschiedenen Aktionen (z. B. Fragen stellen, Arzneimittel empfehlen, Beratungshinweise geben) wählen kann. Durch die Vielfalt der vorgegebenen Gesprächs- und Handlungsmöglichkeiten kann eine „echte“ Beratungssituation gut nachempfunden werden. Im Gegensatz zur analogen Übungsapotheke werden jedoch Kommunikationsfähigkeiten kaum geschult. Durch vorgegebene Fragen muss der Studierende nicht eigenständig formulieren, auch Mimik und Gestik spielen keine Rolle. Stattdessen liegt der Schwerpunkt dieses Lehr- und Lernformates auf der Anwendung des pharmazeutischen Fachwissens für einen individuellen Patienten.

### Patientenorientierte Lehrveranstaltungen in Einzel- oder Kleingruppenunterricht im klinischen Setting

#### Unterricht in der öffentlichen Apotheke.

Die Übungsapotheke bleibt eine Nachstellung der realen Situation mit Laienschauspielern, die die Patienten mimen. Diese erhalten zwar Regieanweisungen, das mögliche Spektrum an unterschiedlichen Verhaltensweisen lässt sich dadurch jedoch nicht authentisch abbilden. So lassen sich kommunikative Schwierigkeiten, wie unklare Ausdrucksweisen oder Missverständnisse, die sich z. B. durch Alter, Krankheit oder fehlende Sprachkenntnisse des Patienten ergeben, durch Laien kaum glaubwürdig darstellen. Eine geeignete Abhilfe für diese Problematik ist die Ausbildung von Pharmaziestudierenden in öffentlichen Apotheken. Bisher bietet kein Universitätsstandort dieses Lehrformat regelhaft im Pharmaziestudium an (Abb. 2), lediglich ein Standort ist in der konkreten Umsetzungsplanung. Mögliche Verhinderungsgründe könnten fehlende personelle Ressourcen und ein hoher organisatorischer Aufwand sein.

#### Unterricht im Krankenhaus mit Patientenkontakt („Bedside Teaching“).

Seit 2012 werden die Studierenden der FAU Erlangen-Nürnberg in einem sogenannten „Bedside-Teaching“-Praktikum auf Krankenhausstationen des Universitätsklinikums Erlangen unterrichtet. Im Zentrum des Praktikums steht das Patientengespräch, welches eigenständig durch die Studierenden als Arzneimittelanamnese- oder Beratungsgespräch durchgeführt wird. Im Unterschied zu didaktisch aufbereiteten Fällen sind echte Patienten und ihre Arzneimitteltherapie in der Regel sehr komplex. Daher bietet sich der Einsatz dieses Lehrformates in höheren Semestern an. Zur Vorbereitung setzen sich die Studierenden intensiv mit dem Patientenfall auseinander, indem sie eine Medikationsanalyse durchführen. Dabei werden alle relevanten Patienteninformationen berücksichtigt, um mögliche arzneimittelbezogene Probleme zu identifizieren und Lösungen zu erarbeiten. Diese werden nachfolgend mit dem ärztlichen oder Pflegepersonal besprochen. Das Praktikum wird von einem klinisch tätigen Apotheker (engl. „Teacher Practitioner“) betreut und in Kleingruppen durchgeführt. Dieses Lehrformat bietet sehr gute Möglichkeiten, um verschiedene klinisch-pharmazeutische Kompetenzen (z. B. individuelles Anwenden des Fachwissens, Kommunikation) zu trainieren. Der Nutzen in der pharmazeutischen und humanmedizinischen universitären Ausbildung konnte wissenschaftlich nachgewiesen werden [23–25]. Es bieten 11 weitere Hochschulstandorte ein „Bedside-Teaching“-Praktikum an (Abb. 2; [14, 26–28]).

### Interprofessionelles Lehren und Lernen

Die Gesundheitsversorgung wird durch eine enge interprofessionelle Zusammenarbeit von Gesundheitsberufen verbessert [29]. Um diese zu fördern, bietet es sich an, interprofessionelles Lehren und Lernen schon in der Ausbildung einzusetzen. Nach einer erfolgten Pilotierung findet seit dem Wintersemester 2015/2016 an der LMU München die berufsübergreifende Ausbildungsveranstaltung „POP Art – Patientenorientierte Pharmazie für (angehende) Ärzte und Apotheker“ statt. Ein Team, bestehend aus 1–2 Pharmaziestudierenden und einem Medizinstudierenden im Praktischen Jahr, verbringt einen halben Tag gemeinsam auf einer Krankenhausstation. Ziel der Veranstaltung ist es, schon früh eine Grundlage für eine erfolgreiche Kommunikation an der Schnittstelle zwischen Medizin und Pharmazie zu legen. Weiterhin soll der klinische Praxisbezug im Studium für die Pharmaziestudierenden erhöht werden und die angehenden Humanmediziner sollen die Möglichkeit haben, ihre praktischen pharmakologischen Kenntnisse zu erweitern [14, 30]. Zurzeit nutzen 10 pharmazeutische Universitätsstandorte ein interprofessionelles Lehrformat in der Ausbildung, wobei der Kurs lediglich an 3 Standorten verpflichtend ist (Abb. 2). Bei den angebotenen Lehrveranstaltungen handelt es sich sowohl um gemeinsames Lernen von Studierenden/Auszubildenden unterschiedlicher Fachrichtungen (5 Standorte) als auch um Unterricht für Pharmaziestudierende durchgeführt durch einen Arzt (4 Standorte). Ein Standort machte keine weiteren Angaben zu den beteiligten Berufsgruppen.

An 2 weiteren Universitäten wird interprofessionelles Lernen im 3. Abschnitt der Pharmazeutischen Ausbildung angeboten (siehe auch Beitrag von Weber et al. in diesem Themenheft).

### Patientenorientierte Prüfungsformen – „objective structured clinical examination“ (OSCE)

Um klinische Fähigkeiten und Kompetenzen prüfen zu können, müssen neue Prüfungsformen entwickelt und etabliert werden, die über eine reine traditionelle Wissensabfrage hinausgehen. International wie auch im deutschen Humanmedizinstudium haben sich zu diesem Zweck OSCE-Prüfungen etabliert [31, 32]. OSCE-Prüfungen erlauben die Beurteilung von klinischen Kompetenzen (z. B. Kommunikation, klinisches Anwendungswissen, Problemlösungs- und berufliches Urteilsvermögen; [33]). Bei dieser Prüfungsform wird mithilfe eines (Laien‑)Schauspielers eine Aufgaben‑/Problemsituation aus dem klinischen Alltag nachgestellt, die vom Prüfling zu bewältigen ist. Die Bewertung wird durch eine dritte Person anhand eines Fragebogens durchgeführt, in dem neben angewandtem Fachwissen auch Soft Skills bewertet werden. In Deutschland werden OSCE verpflichtend an 2 pharmazeutischen Universitätsstandorten eingesetzt. 2 weitere Standorte bieten OSCE optional an (Abb. 2). OSCE werden meist in einer Art Zirkeltraining durchgeführt, um mehrere Aufgabensituationen beurteilen zu können [23, 24, 28].

### Arzneimittelinformation

Da die Information und Beratung in der Apotheke per Definition eine pharmazeutische Tätigkeit ist, ergibt sich die Notwendigkeit zur guten Praxis der Arzneimittelinformation als Verpflichtung für jeden Apotheker [34]. Zur Sicherung der Qualität des Prozesses ist die Fähigkeit zu einer individualisierten, wissenschaftlich fundierten Arzneimittelinformation, inklusive der Bewertung der Rechercheergebnisse, essenziell [35, 36]. Abgesehen von der rechtlichen Verpflichtung zur Arzneimittelinformation, stellen das Auffinden und die Interpretation von validen, evidenzbasierten Quellen eine grundlegende Voraussetzung für die gute Praxis der Ausübung von klinisch-pharmazeutischen Tätigkeiten dar. Die Arzneimittelinformation ist somit für das Fach Klinische Pharmazie und die darin angebotenen Lehrformate (z. B. das fallbezogene Lernen, das Ausführen einer Patientenfall-basierten Medikationsanalyse) unabdingbar. Daher sollte die Recherche von und der Umgang mit pharmazeutischer Fachliteratur in der Lehre im Fach Klinische Pharmazie integriert sein [13].

Im Rahmen einer Förderung durch die Lesmüller-Stiftung und die Bayerische Landesapothekerkammer wird an den Bayerischen Universitäten Regensburg (seit 2001), Würzburg (seit 2003) und Erlangen (seit 2006) ein festes Stundendeputat für Arzneimittelinformation für alle Pharmaziestudierenden angeboten. Dies umfasst neben der Lehre der theoretischen Grundlagen der Arzneimittelinformation in Vorlesungen und Seminaren auch die fallbasierte, patientenorientierte Bearbeitung von Arzneimittelinformationsanfragen. So werden an der FAU Erlangen-Nürnberg in einem studentischen Kleingruppenpraktikum bereits beantwortete Echtanfragen aus der regionalen Arzneimittelinformationsstelle am Universitätsklinikum Erlangen in Zweiergruppen recherchiert und die Beantwortung der Fragestellung schriftlich ausgearbeitet. Zusätzlich wird von jeder Zweiergruppe der Rechercheweg in einer Präsentation ausführlich dargestellt und den Kommilitonen vorgestellt. Auf diese Weise lernen die Pharmaziestudierenden durch Eigenleistung nicht nur verschiedene pharmazeutische Fachinhalte, sondern durch das selbstständige Recherchieren und Interpretieren von Informationen auch die Grundlagen des fachlich-praktischen Umgangs mit Arzneimittelinformationen und der evidenzbasierten Medizin kennen.

## Fazit und Ausblick

**Abb. 3:**
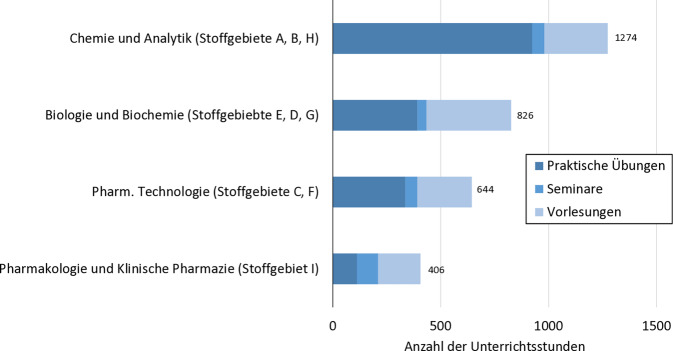
Anzahl der Unterrichtsstunden in den Stoffgebieten des Pharmaziestudiums gemäß aktueller Approbationsordnung für Apotheker (AappO). Stoffgebiet *A* Allgemeine Chemie der Arzneistoffe, Hilfsstoffe und Schadstoffe, Stoffgebiet *B* Pharmazeutische Analytik, Stoffgebiet *C* Wissenschaftliche Grundlagen, Mathematik und Arzneiformenlehre, Stoffgebiet *D* Grundlagen der Biologie und Humanbiologie, Stoffgebiet *E* Biochemie und Pathobiochemie, Stoffgebiet *F* Pharmazeutische Technologie und Biopharmazie, Stoffgebiet *G* Biogene Arzneistoffe, Stoffgebiet *H* Medizinische Chemie und Arzneistoffanalytik, Stoffgebiet *I* Pharmakologie und Klinische Pharmazie (nicht berücksichtigt ist Stoffgebiet *K* Wahlpflichtfach mit 112 h; [2]). (Quelle: eigene Abbildung)

Durch den Wandel des Berufsbildes von Apothekern sowohl in der Krankenhaus- als auch in der öffentlichen Apotheke nimmt die Bedeutung einer patientenorientiert ausgeübten Pharmazie sehr deutlich zu. Dabei nimmt der Apotheker als integraler Bestandteil des heilberuflichen interprofessionellen Netzwerkes eine wichtige Mitverantwortung zur Verbesserung der Arzneimitteltherapiesicherheit und zur Durchführung einer optimierten Arzneimitteltherapie am und durch den Patienten, wahr [5, 8–10]. Dem stark veränderten Berufsanforderungsprofil und den gestiegenen klinisch-patientenorientierten Kompetenzanforderungen an den Apothekerberuf steht in der aktuellen universitären Apothekerausbildung in Deutschland ein Unterrichtsstundendeputat von 12,4 % der Gesamtausbildungsstunden für die 2 Fächer des Stoffgebietes I (Pharmakologie und Klinische Pharmazie) entgegen ([2]; Abb. 3). Es ist daher sehr offensichtlich notwendig, eine Aktualisierung der Apothekerausbildung in Deutschland anzustreben [15, 16]. Die Bundesapothekerkammer und andere Organisatoren und Fachverbände haben aufgrund dessen im Jahr 2022 ein Positionspapier zur Novellierung der Approbationsordnung für Apotheker ausgearbeitet [15]. Darin heißt es, dass die Fächer Klinische Pharmazie und Pharmakologie zeitlich und inhaltlich erweitert werden sollen. Beide Fächer sind auch geeignet, um den Anspruch einer verstärkten interprofessionellen Ausbildung von Studierenden der Pharmazie und Humanmedizin umzusetzen. Das Fach Klinische Pharmazie wird in diesem Reformvorschlag unter anderem durch die verpflichtende Einführung eines Moduls „Pharmazeutische Betreuung“ gestärkt, dass regelhaft ein umfassendes klinisch-pharmazeutisches Praktikum auf einer Krankenhausstation oder im ambulanten Bereich vorsieht.

Aufgrund unserer Literaturrecherche und der durchgeführten Ad-hoc-Umfrage an allen 22 deutschen Universitätsstandorten zu den angebotenen patientenorientierten Lehrformaten im Fach Klinische Pharmazie lässt sich feststellen, dass das geforderte fallbasierte, theoretische patientenorientierte Lehren und Lernen gemäß Standard 6 der DPhG-Fachgruppe Klinische Pharmazie [13] nahezu flächendeckend umgesetzt wird. Deutlich wird aber auch, dass nur an 6 von den 19 antwortenden Ausbildungsstandorten 4 oder mehr patientenorientierte Lehrformate praktiziert werden (Abb. 1). Aus den insgesamt 7 abgefragten patientenorientierten Lehrformaten heben sich die Formate „Bedside Teaching“ mit 12 Nennungen und interprofessionelle Lehre mit 10 Nennungen hervor. Mehrheitlich werden beide Formate optional angeboten (Abb. 2). Der zeitliche Umfang ist beschränkt, was angesichts stark begrenzter personeller Ressourcen nicht überrascht. Vielfach wird das „Bedside Teaching“ zumindest zum Teil ehrenamtlich oder durch Lehraufträge von Krankenhausapothekern geleistet. Interprofessionelles Lehren bzw. Lernen wird an 10 Universitätsstandorten im Fach Klinische Pharmazie in zeitlich begrenztem Umfang praktiziert.

Das sich bietende Gesamtbild zeigt eine insgesamt sehr heterogen praktizierte patientenorientierte Lehre im Fach Klinische Pharmazie. Dies betrifft sowohl die Art des Lehrangebotes als auch die Verpflichtung zur Teilnahme für die Studierenden. Positiv festzuhalten ist, dass es Leuchtturmstandorte gibt, die trotz begrenzter Ressourcen mit zum Teil Drittmittel-finanziertem und/oder ehrenamtlichem Einsatz von Lehrpersonen den Studierenden bereits heute patientenorientierte Lehrformate anbieten. Eine zukünftige universitäre Ausbildung sollte in jedem Fall mit einer suffizienten Lehrpersonalausstattung zeitlich deutlich umfassendere und verpflichtende, patientenorientierte Lehrformate im Fach Klinische Pharmazie vorsehen. Hierzu zählen insbesondere auch Lehrformate, die das interprofessionelle Lehren und Lernen fördern, z. B. auf Krankenhausstationen und im ambulanten Versorgungsbereich.

Die Notwendigkeit, durch eine „Novellierung der Approbationsordnung für Apotheker“ zu einer inhaltlichen Neuausrichtung und neuen Schwerpunktsetzung und damit auch Ressourcenallokation in der Apothekerausbildung in Deutschland zu kommen, ist angesichts des heutigen beruflichen Anspruches an Apotheker und ihre Berufsausübung in der ambulanten und stationären Patientenversorgung offensichtlich.

## Supplementary Information

Im Onlinematerial 1 wird die Suchstrategie zur strukturierten Literaturrecherche dargestellt.

Im Onlinematerial 2 ist der Fragebogen zur Abfrage des Einsatzes von patientenorientieren Lehrformaten inkl. Art und Umfang an allen deutschen Universitätsstandorten dargestellt.
