# Supplementary material for: Patient-oriented teaching formats in clinical pharmacy—status quo in German university pharmacist education
Source: Bundesgesundheitsblatt Gesundheitsforschung Gesundheitsschutz. 2025 Mar 20;68(5):495–502. [Article in German] doi: 10.1007/s00103-025-04036-2 (PMC12075349; doi:10.1007/s00103-025-04036-2)
Supplement: Supplementary file 1 — Im Onlinematerial 1 wird die Suchstrategie zur strukturierten Literaturrecherche dargestellt. [file 103_2025_4036_MOESM1_ESM.pdf]

Onlinematerial 1 zu

Patientenorientierte Lehrformate in der Klinischen Pharmazie – zum Status Quo in der deutschen universitären Apothekerausbildung

Frank Dörje, Mirjam Gnadt, Jacqueline Bauer, Monika Dircks

zu Methodik: Strukturierte Literaturrecherche

- **Recherchierter Zeitraum:** letzten 20 Jahre

- **Primäre Suchbegriffe:**
  - Patientenorientierte Pharmazie
  - Klinische Pharmazie UND
    - Patientenorientierte Lehre
    - Patientenorientierte Lehrformate
    - fallbasiertes Lernen
    - fallbasierte Lehre

- **Recherchierte Quellen: Deutschsprachige Fachzeitschriften/Websites/wissenschaftliche Suchmaschine**
  - Krankenhauspharmazie
  - Deutsche Apothekerzeitung
  - Pharmazeutische Zeitung
  - Websites deutscher Hochschulstandorte mit Studiengang Pharmazie
  - Google Scholar

- **Sekundäre Suchbegriffe** *(Anm.: Suchbegriffe, die sich nach der primären Recherche ergeben haben)***:**
  - Ausbildungsapotheke
  - Virtuelle (Ausbildungs-)Apotheke
  - IPSTA (Interprofessionelle Ausbildungsstation)
  - MyDispense
  - OSCE (objective structured clinical examination)-Prüfung
