# Supplementary material for: Patient-oriented teaching formats in clinical pharmacy—status quo in German university pharmacist education
Source: Bundesgesundheitsblatt Gesundheitsforschung Gesundheitsschutz. 2025 Mar 20;68(5):495–502. [Article in German] doi: 10.1007/s00103-025-04036-2 (PMC12075349; doi:10.1007/s00103-025-04036-2)
Supplement: Supplementary file 2 — Im Onlinematerial 2 ist der Fragebogen zur Abfrage des Einsatzes von patientenorientieren Lehrformaten inkl. Art und Umfang an allen deutschen Universitätsstandorten dargestellt. [file 103_2025_4036_MOESM2_ESM.pdf]

Apotheke des Universitätsklinikums Erlangen/Friedrich-Alexander-Universität Erlangen-Nürnberg
Prof. Frank Dörje, MBA; Dr. Mirjam Gnadt; Monika Dircks

Stand: 27.10.2024

Rücksendung bitte an frank.doerje@uk-erlangen.de

Ad-hoc Umfrage - Deutsche Universitätsstandorte, Fach Klinische Pharmazie

Status-Quo: Patienten-orientierte Lehrformate

Geben Sie Ihre Antworten bitte in die farbig markierten Felder ein.

Universitätsstandort:	
Ansprechpartner/in:	

Welche patienten-orientierten Lehrformate werden an Ihrem Standort im Fach Klinische Pharmazie angeboten?

A Theoretische patienten-orientierte Lehrformate im universitären Setting

A1. Theoretische Patientenfälle, die in Einzelarbeit oder Kleingruppen bearbeitet werden:

Bitte ankreuzen (X)	ja		nein	

Falls ja:

Bitte ankreuzen (X)	Pflichtveranstaltung:		Freiwilliges Angebot:	
Freitext	Zeitlicher Umfang:	(aus Sicht des Studenten; z.B. in SWS, einmalig 4h, pro Woche 3h im 7. Fachsemester)		
Freitext	Betreuung durch:	(z.B. Professor/in, Doktorand, klinisch-tätiger Apotheker)		
Freitext	Ggf. weitere Informationen:			

A2. Virtuelle Patientenfallbearbeitung, die in Einzelarbeit oder Kleingruppen bearbeitet werden (z.B. Casus®):

Bitte ankreuzen (X)	ja		nein	

Falls ja:

Bitte ankreuzen (X)	Pflichtveranstaltung:		Freiwilliges Angebot:	
Freitext	Zeitlicher Umfang:	(aus Sicht des Studenten; z.B. in SWS, einmalig 4h, pro Woche 3h im 7. Fachsemester)		
Freitext	Betreuung durch:	(z.B. Professor/in, Doktorand, klinisch-tätiger Apotheker)		
Freitext	Ggf. weitere Informationen:			

A3. Übungs-Offizin-Apotheke mit Hilfe von (Laien-)Schauspielern:

Bitte ankreuzen (X)	ja		nein	

Falls ja:

Bitte ankreuzen (X)	Pflichtveranstaltung:		Freiwilliges Angebot:	
Freitext	Zeitlicher Umfang:	(aus Sicht des Studenten; z.B. in SWS, einmalig 4h, pro Woche 3h im 7. Fachsemester)		
Freitext	Betreuung durch:	(z.B. Professor/in, Doktorand, klinisch-tätiger Apotheker)		
Freitext	Ggf. weitere Informationen:			

A4. Virtuelle Übungs-Offizin-Apotheke (z.B. MyDispense®):

Bitte ankreuzen (X)	ja		nein	

Falls ja:

Bitte ankreuzen (X)	Pflichtveranstaltung:		Freiwilliges Angebot:	
Freitext	Zeitlicher Umfang:	(aus Sicht des Studenten; z.B. in SWS, einmalig 4h, pro Woche 3h im 7. Fachsemester)		
Freitext	Betreuung durch:	(z.B. Professor/in, Doktorand, klinisch-tätiger Apotheker)		
Freitext	Ggf. weitere Informationen:			

B Patienten-orientierte Lehrveranstaltungen in Einzel- oder Kleingruppen im klinischen Setting (Krankenhausstation/"reale" Offizin-Apotheke)

B1. Unterricht im Krankenhaus mit Patientenkontakt ("bedside teaching")

Bitte ankreuzen (X)	ja		nein	

Falls ja:

Bitte ankreuzen (X)	Pflichtveranstaltung:		Freiwilliges Angebot:	
Freitext	Zeitlicher Umfang:	(aus Sicht des Studenten; z.B. in SWS, einmalig 4h, pro Woche 3h im 7. Fachsemester)		
Freitext	Betreuung durch:	(z.B. Professor/in, Doktorand, klinisch-tätiger Apotheker)		
Freitext	Ggf. weitere Informationen:			

B2. Training in "realer" Offizin-Apotheke mit "echten" Patienten

Bitte ankreuzen (X)	ja		nein	

Falls ja:

Bitte ankreuzen (X)	Pflichtveranstaltung:		Freiwilliges Angebot:	
Freitext	Zeitlicher Umfang:	(aus Sicht des Studenten; z.B. in SWS, einmalig 4h, pro Woche 3h im 7. Fachsemester)		
Freitext	Betreuung durch:	(z.B. Professor/in, Doktorand, klinisch-tätiger Apotheker)		
Freitext	Ggf. weitere Informationen:			

B3. Interprofessionelle Lehre im stationären Setting

Bitte ankreuzen (X)	ja		nein	

Falls ja:

Bitte ankreuzen (X)	Pflichtveranstaltung:		Freiwilliges Angebot:	
Freitext	Zeitlicher Umfang:	(aus Sicht des Studenten; z.B. in SWS, einmalig 4h, pro Woche 3h im 7. Fachsemester)		
Freitext	Betreuung durch:	(z.B. Professor/in, Doktorand, klinisch-tätiger Apotheker)		
Freitext	Teilnehmende Berufsgruppe:	(z.B. Medizinstudenten, Physiotherapeuten, Pflegeschüler)		
Freitext	Ggf. weitere Informationen:			

C Weiteres

C1. Werden OSCE (Objective structured clinical examination) an ihrem Standort eingesetzt?

Bitte ankreuzen (X)	ja		nein	

Falls ja:

Bitte ankreuzen (X)	Pflichtveranstaltung:		Freiwilliges Angebot:	
Bitte ankreuzen (X)	"Echte" Patienten		(Laien-)Schauspieler	
Freitext	Zeitlicher Umfang:	(aus Sicht des Studenten; z.B. in SWS, einmalig 4h, pro Woche 3h im 7. Fachsemester)		
Freitext	Betreuung durch:	(z.B. Professor/in, Doktorand, klinisch-tätiger Apotheker)		
Freitext	Ggf. weitere Informationen:			

C2. Wie wird an ihrem Standort Arzneimittelinformation gelehrt?

Bitte ankreuzen (X)	Vorlesung:		Übungen:	
Freitext	Ggf. weitere Informationen:			

C3. Sind patienten-orientierte Lehrangebote in Planung oder mussten welche in der Vergangenheit eingestellt/gekürzt werden?
Bitte erläutern sie kurz und geben ggf. Gründe an:

